# HBV‐integrated local genomic alterations reveal multicentric independent occurrences of multifocal HCC

**DOI:** 10.1002/ctm2.1313

**Published:** 2023-06-29

**Authors:** Hao Zou, Yinan Wang, Lianfang Lu, Ke Yao, Chang Wang, Kai Ma, Chengzhan Zhu, Zhongyi Guo, Yujie Feng, Zehua Wu, Mengqi Song, Bin Zhou, Xiao Hu, Bing Han, Weidong Guo, Fabo Qiu, Bingyuan Zhang, Xingsi Qi, Xiaowei Wang, Mengyao Wang, Guangze Pan, Qixuan Sun, Jingyu Cao, Song Gong, Zicheng Zhao, Chuandong Sun, Shichun Lu, Lantian Tian

**Affiliations:** ^1^ Department of Hepatopancreatobiliary Surgery the Affiliated Hospital of Qingdao University Qingdao China; ^2^ Department of Obstetrics and Gynecology Peking University Shenzhen Hospital Shenzhen China; ^3^ School of Medicine Southern University of Science and Technology Shenzhen China; ^4^ Department of Obstetrics the Affiliated Hospital of Qingdao University Qingdao China; ^5^ Department of Gynecology the Affiliated Hospital of Qingdao University Qingdao China; ^6^ Department of Gastroenterology the Affiliated Hospital of Qingdao University Qingdao China; ^7^ Shenzhen Byoryn Technology Co., Ltd Shenzhen China; ^8^ College of Medicine and Biological Information Engineering Northeastern University Shenyang China; ^9^ Department of Trauma Surgery Tongji Trauma Center Tongji Hospital Tongji Medical College Huazhong University of Science and Technology Wuhan China; ^10^ Key Laboratory of Digital Hepatobiliary Surgery Chinese People's Liberation Army General Hospital Beijing China

Dear Editor,

We sampled two different HCC tumours from a patient with HBV infection and found different HBV integration events, suggesting multicentric occurrence (MO) rather than intrahepatic metastasis (IM).

HCC is the third leading cause of cancer‐related deaths worldwide. Half of the HCC is associated with HBV infection, frequently accompanied by HBV DNA integration into the host genome.^1^ Multifocal HCC with high incidence can arise either from IM or MO. Differentiation between IM and MO is required, as the treatment options and prognosis vary between these two types. In the absence of a standard to distinguish the clonal origin, integration of HBV provides molecular evidence of clonal origin. HBV DNA might integrate into the host genome long before hepatocarcinogenesis in chronic HBV patients, eventually leading to HCC. As such, the HBV‐integrated local haplotype would be specific to each tumour clone and could discriminate between the IM and MO.^2^ IM implies that all tumour cells develop from the first cancer cell, thus having at least one shared HBV integration event. In contrast, HBV integration events occurred independently in different tumour cells in MO HCC. Analysis of the integration patterns of HBV can determine the clonal origin. In contrast to well‐studied IM, the characteristics of HBV independent integration in MO remain underexplored.^3^

To find the rare independent HBV integration event more economically and efficiently, we first randomly selected one lesional sample from each multifocal HBV‐positive HCC patient (*n* = 7; Table S1) for sequencing and found that only one sample (T1) with the most SVs had multiple HBV integration sites (Figure S1 and Tables S2 and S3). Compared to patients with no integration site (or only one), the independent event of HBV integration most likely occurred in the multifocal patient who provided the T1 sample. So we sequenced one more tumour sample (T2) from another lesion of this patient and finally obtained short and long‐read sequencing data from T1 and T2. We refactored the FuseSV^4^ to make it available for long‐read sequencing data and reconstructed the haplotype‐resolved structures at the HBV integration loci to further understand the origin of these two foci (see Supplementary Methods for details; Figure 1).

**FIGURE 1:**
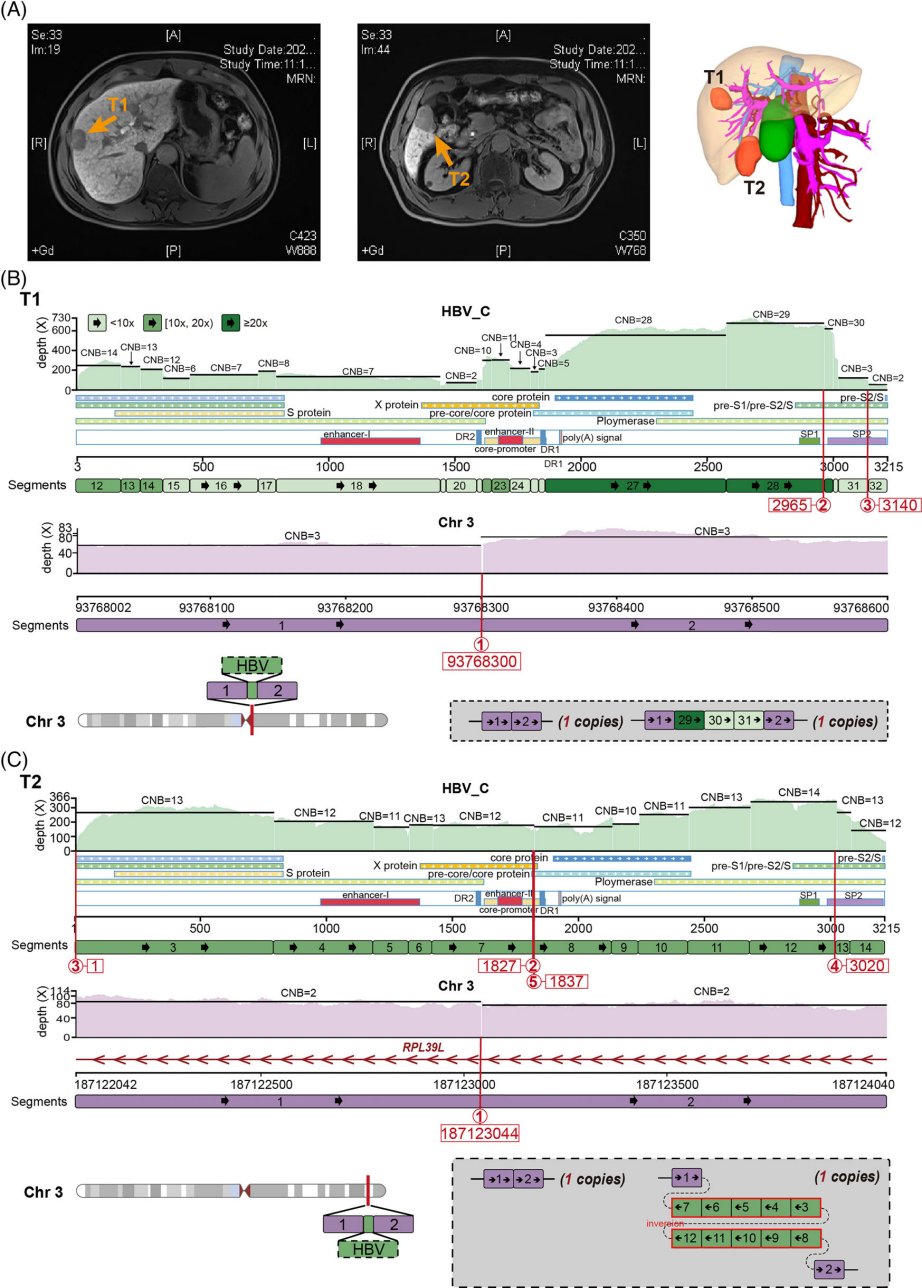
The local haplotype of HBV‐integrated genomic region in chr3 of T1 and T2. (A) The magnetic resonance image of T1 (left) and T2 (right) and the 3‐D visualisation reconstruction of T1 and T2. Yellow arrows indicate the tumour lesions. (B) Presentative local haplotype at HBV integration sites on chr3 of T1. Top: constructed HBV_C genome is segmented (12∼32). The sequencing depth spectrum is displayed with balanced copy numbers of segments. Black lines denote the average depth of segments. Breakpoints are noted by circled numbers. Middle: human genomic region of chr1 and chr7 flanking HBV integrations are divided into segments (1∼2) by viral insertions. Bottom left: schematic diagram of local haplotype and its location on chr3. Bottom right: resolved alleles of local haplotype are indicated as coloured segments connected string with copy times. (C) Presentative local haplotype at HBV integration sites on chr3 (*RPL39L* gene) of T2.

There are no identical integration loci and local haplotypes between these foci (Table S2; Figures 1, S2 and S3). Eleven and two integration loci were identified in T1 and T2, respectively. T1 and T2 each had one HBV fragment inserted on chr3q (Figure 1B and C). The integration locus of T1 was located in the centromere region on chr3 (3q11.1). The inserted HBV DNA segments (‘29’ to ‘31’) contained a part of the PreS2 promoter. Conversely, the HBV segments, inserted at the intron 2 of ribosomal protein L39 like (*RPL39L*) gene on chr3q27.3 of T2, were absent of a part of the PreS2 promoter. Moreover, T1 had seven other HBV integration loci on chr1, 5, 7 and 8 (Figures S2 and S3). Specifically, a fusion of chr1 and 7 were linked by an HBV bridge (Figure S4). This HBV ‘bridge’ had a fold‐back inversion where duplicated segments are arranged head to head. Through this ‘bridge’, the short arm of chr7 was concatenated with chr1q in the reverse orientation, replacing the tail (3‐prime) of 1q (Figure S2A and B). This process generated one hybrid chromosome, whose long arm consisted of a region from 1q and the short arm of chr7. For T1, the two remaining integration sites were inserted at exon 1 of the *TERT* hotspot on chr5 and intron 1 of the testis development‐related protein (*TDRP*) gene on chr8, respectively (Figure S3). Both were located near telomeres (5p15.33 and 8p23.3), and both inserted HBV segments carried the core promoter, enhancer II and PreS1 promoter. Meanwhile, we obtained short‐read sequencing data from four foci in one HBV‐HCC patient (47‐year‐old male).^3^ Unlike T1 and T2, these four foci (C1‐4) shared one HBV integration event, which formed a hybrid chromosome between chr1 and chr8 (Figure S5). This revealed a monoclonal origin of this multifocal HCC.

**FIGURE 2:**
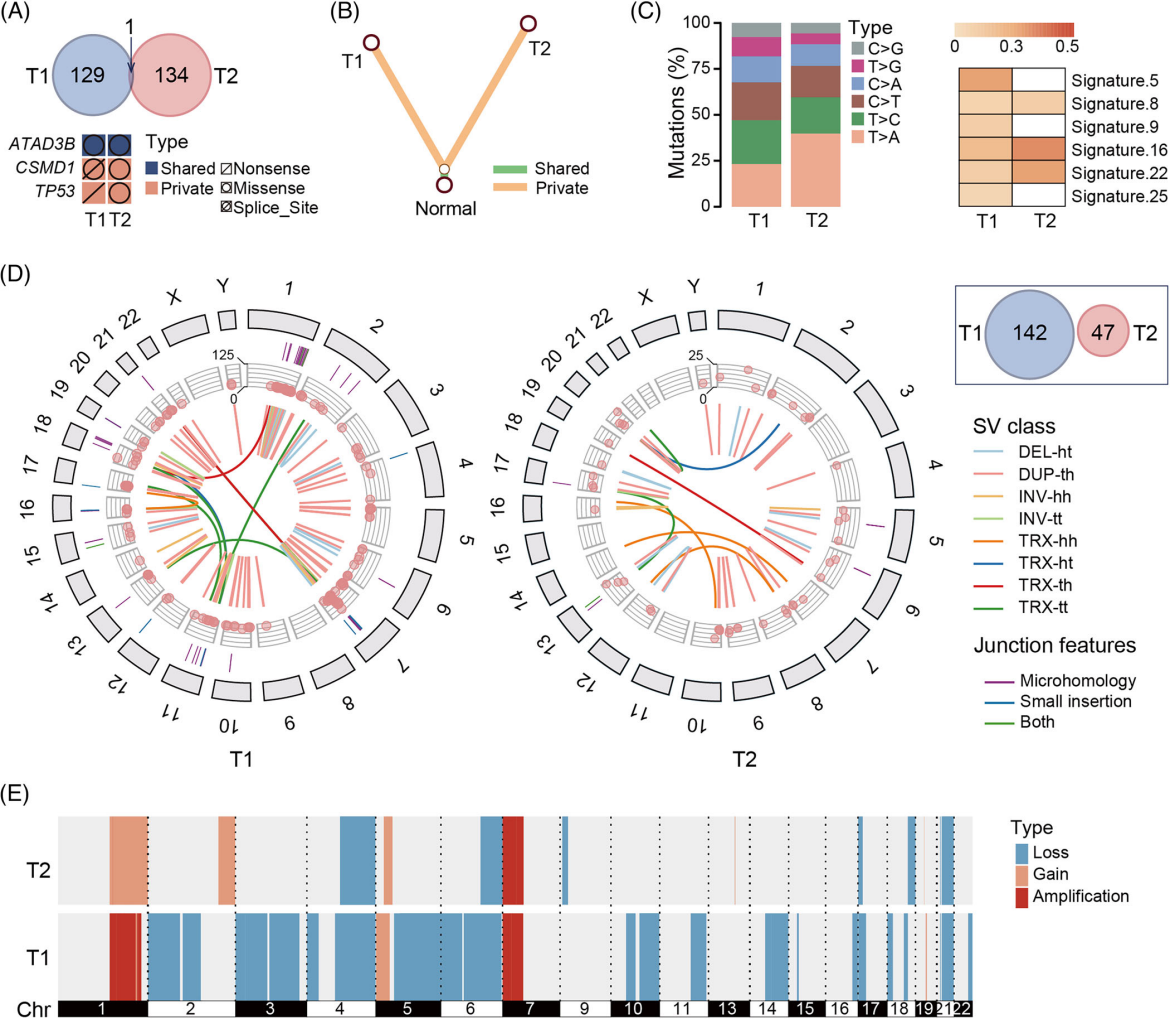
Detection of multiple hepatocellular carcinomas (HCC) in the patient. (A) The shared and private somatic SNVs between T1 and T2. (B) The phylogenetic tree was constructed based on the present/absence status of all mutations between T1 and T2. (C) The mutation signature of T1 and T2. (D) The SVs circular view and Venn of T1 and T2. The outer ring shows the ideogram of the human autosomes, labelled with chromosome numbers. The segments represent the microhomology and small insertion. The point tracks show the number of supporting reads for rearrangement. The different coloured links represent the different rearrangements. (E) CNV profile of T1 and T2. DEL‐ht: head‐to‐tail deletion; DUP‐th: tail to head duplication; INV‐hh: Head‐to‐head inversion; INV‐tt: tail to tail inversion; TRX‐hh: head‐to‐head translocation; TRX‐ht: head‐to‐tail translocation; TRX‐th: tail‐to‐head translocation; TRX‐tt: tail‐to‐tail translocation.

The integration patterns of HBV were different in IM and MO. The HBV‐integrated local haplotype of the monoclonal origin of multifocal HCC was shared among lesions, whereas the completely different local haplotypes of T1 and T2 suggested that these two lesions were MO, of which HBV integration events occurred independently.

Moreover, the substantial heterogeneity of SNVs and SVs also supported that T1 and T2 were MO (Figure 2; Tables S3–S5). We identified 130 and 134 somatic SNVs in T1 and T2, respectively. Only one SNV (c.C1915T in the *ATAD3B* exon16) was shared by two lesions. T1 contained a nonsense mutation in *TP53* (p.E336*; neutral), while T2 possessed a missense mutation in the driver gene *TP53* (p.S215N; pathogenic). The phylogenetic tree, constructed based on the present/absence status of all mutations, showed that T1 and T2 were distant. No shared clusters of tumours were detected between T1 and T2 by PyClone.^5^ The proportions of six types of mutations varied between the two lesions. Numerous WGS studies revealed that different lesions with nearly no common mutations suggested independent tumour origins, whereas IM tumours shared 8%‐97% mutations and often had the same mutation pattern in the driver gene.^6^ The different SV breakpoints also indicated multiple occurrences.^7^

The main drawback is that we only found one patient with independent integration events. This would weaken the reliability of the conclusion and cannot accurately character more complex cases (e.g., both identical and different integrations between multiple foci in one patient).

In conclusion, we hypothesise that analysis of HBV integration in patients with multifocal HCC may help distinguish MO from IM, a determination that has significant clinical implications.

## CONFLICT OF INTEREST STATEMENT

Author Mengyao Wang, Guangze Pan and Zicheng Zhao are employed by Shenzhen Byoryn Technology Co., Ltd. The remaining authors declare that they have no conflict of interest.

## Supporting information

Supplementary Methods. The detailed materials and methods in the study.
Figure S1. Characterisations of SVs and HBV integration of seven patients.
Figure S2. The local haplotype of HBV‐integrated genomic region in chr1 and chr7 of T1.
Figure S3. Presentative local haplotype at HBV integration loci on chr5 (*TERT*) and chr8 (*TDRP*) of the T1 sample.
Figure S4. Eight long PacBio reads supported the fusion of chr1 and chr7 linked by an HBV bridge.
Figure S5. The local haplotype of HBV‐integrated genomic region of C1‐4.
Figure S6. Sequence alignment between three HBV subtypes.

Table S1. Clinical characteristics of patients with hepatocellular carcinoma.
Table S2. Details of HBV integrations.
Table S3. Details of SVs from short‐read sequencing data.
Table S4. Details of SNVs and InDels from short‐read sequencing data.
Table S5. Details of CNVs from short‐read sequencing data.
Table S6. Quality control for short‐read sequencing data.
Table S7. Purity and ploidy of tumour samples.

## Data Availability

The raw sequencing data reported here have been deposited in the China National GeneBank DataBase (CNGBdb) with accession number CNP0003155.
