# Supplementary material for: HBV‐integrated local genomic alterations reveal multicentric independent occurrences of multifocal HCC
Source: Clin Transl Med. 2023 Jun 29;13(6):e1313. doi: 10.1002/ctm2.1313 (PMC10309081; doi:10.1002/ctm2.1313)
Supplement: Supplementary file 1 — Supplementary Methods. The detailed materials and methods in the study. Figure S1. Characterisations of SVs and HBV integration of seven patients. Figure S2. The local haplotype of HBV‐integrated genomic region in chr1 and chr7 of T1. Figure S3. Presentative local haplotype at HBV integration loci on chr5 (TERT) and chr8 (TDRP) of the T1 sample. Figure S4. Eight long PacBio reads supported the fusion of chr1 and chr7 linked by an HBV bridge. Figure S5. The local haplotype of HBV‐integrated genomic region of C1‐4. Figure S6. Sequence alignment between three HBV subtypes. [file CTM2-13-e1313-s002.docx]

**Supplemental Materials**

**1 Supplementary Methods**

**1.1 Sample selection**

We recruited seven patients with HBV-HCC (age range: 55-73; Male/female: 6/1) and collected eight tumor tissues and their paired normal tissues from these patients in the Affiliated Hospital of Qingdao University. Two of these tumor tissue samples (T1 and T2) were from two lesions of the same patient. The clinical diagnosis was verified by cytopathology. The Ethics Committee of the Affiliated Hospital of Qingdao University approved the study (approval no. QYFYWZLL27045). All patients provided signed informed consent. We carried out the experiments according to the guidelines released by the National Health and Family Planning Commission of the People’s Republic of China.

**1.2 Whole-genome sequencing (WGS) for short and long read sequencing**

DNA was extracted from tissue samples using the QIAamp DNA mini kit (Qiagen) according to the manufacturer’s instructions. DNA integrity, purity and concentration were assessed by agarose gel electrophoresis, the NanoDrop2000 spectrophotometer, and the Qubit 2.0 fluorimeter (Thermo Fisher Scientific). Qualified DNA samples were used for library construction, which was performed as previously described.^1^ We performed WGS for all samples on the MGISEQ-2000 platform with 2×150 bp paired-end reads (BGI) and two tumor samples (T1 and T2) using long PacBio read sequencing platform with hifi mode (PacBio). Sequencing data from paired tissue samples were used to assess for germline mutations in order to better identify true somatic mutations. Moreover, we collected WGS data (Illumina Hiseq 2000 platform with 2×101 bp paired-end reads) from four foci in one HBV-HCC patient (47-year-old male) from Tongji Hospital.

**1.3 Somatic mutation calling and analyzing based on short read sequencing data**

After obtaining raw short read sequencing data, SOAPnuke^2^ (http://soap.genomics.org.cn/) was used to remove adapters and filter low-quality reads. Clean reads were aligned to the human reference genome (hg38) using BWA^3^ (v0.7.17-r1188) with the default parameters. The duplicate mark was performed with Picard (http://broadinstitute.github.io/picard/), achieving an average sequencing depth of 71× and 45× for tumors and normal samples, respectively (Table S6). Somatic single nucleotide variants (SNV) and somatic small insertions and deletions were called by MuTect2 and annotated by the Genome Analysis Toolkit Funcotator^4^ (GATK v4.2.0.0) with default parameters. CalculateContamination and LearnReadOrientationModel of GATK were used to assess cross-sample contamination and read orientation bias to filter variants, respectively. Variants outside the exonic or splicing region were filtered out. Variants with frequency greater than 1% were filtered out (Table S4). IntOGen^5^ (https://www.IntOGen.org/) offered HCC driver genes in the International Cancer Genome Consortium (ICGC), The Cancer Genome Atlas (TCGA), Pan-Cancer Analysis of Whole Genomes (PCAWG), Asan Medical Center-Korea (AMCK) and Hartiwig cohorts (n=1616). The phylogenetic tree and the shared and private SNVs between T1 and T2 were analyzed and visualized by MesKit^7^ (v1.1.0). To determine the contribution of 30 known mutational signatures recorded by the COSMIC^8^ (<https://cancer.sanger.ac.uk/cosmic/signatures>), we used the R package DeconstructSigs^9^ (v1.8.0) to T1 and T2. The dominant signature of each sample denotes the signature with the highest proportion of the contribution.

**1.4 Structural variant (SV) calling and analyzing based on short read sequencing data**

SvABA^10^ (v1.1.0) was applied to detect structural variations with short read sequencing data, and SV types were annotated by a Python script (<https://github.com/paprikachan/SVAS/blob/master/scripts/parse_svaba.py>). SVs with the total number of split reads ≥ 6 were retained. We use the R package ggbio^11^ (v2.11) to visualize DNA structural rearrangements.

**1.5 Copy number calling based on short read sequencing data**

We employed patchwork^12^ to perform allele-specific copy number variant (CNV) in HCC short read sequencing data. CNVs with the number of SNP in each region ≥ 10 were retained (Table S5). The CNV profiles of T1 and T2 were visualized by MesKit and the distribution of the copy number along the HBV-integrated chromosomes in T1 and T2 was visualized by Oviz-Bio^6^.

**1.6 HBV integrations in the host genome**

Based on short read sequencing data, HBV integrations in the host genome were identified by SurVirus^13^, which can provide precise integration loci in the host human genome and the virus genome. The virus genome database consisted of the eight most common HBV subtype genomes (AF090842.1 for genotype A, AB033554.1 for genotype B, AB014381.1 for genotype C, M32138.1 for genotype D, AB032431.1 for genotype E, AB036910.1 for genotype F, AB064310.1 for genotype G, and AY090454.1 for genotype H). To reconstruct the complex integrated local genomic alterations of HBV, we aligned long PacBio reads (40×) to the reference combined by hg38 and eight HBV references with pbmm2^14^ (v2.14).

Before reconstructing the HBV-integrated local haplotype, we noticed that two junctions contained loci from HBV B and E and the other seven contained loci from HBV C. So we used CLUSTALW (<https://myhits.sib.swiss/cgi-bin/clustalw>) to align these two genotypes and HBV C. Aligned sequences were visualized by ESPript^15^ (v3.0). Sequence alignment showed high sequence similarity near the integration loci between these two genotypes and HBV C (Figure S6). When we used AB014381.1 (HBV C) as the only HBV reference genome, the same junctions were detected. Subsequently, we combined the HBV integrations detected from short and long reads, refactored the FuseSV^16^ to make it available for long read sequencing data, and reconstructed the HBV-integrated local haplotype based on AB014381.1. Local haplotypes incorporated with HBV were visualized using FuseSV and adapted using Adobe Illustrator (v2021, Adobe).

**1. 7 Clonal Evolution Prediction**

The purity and ploidy of tumor tissue were determined on the copy number of the DNA segment and the allele fraction values of the somatic SNVs by ABSOLUTE^17^ (Table S7). The cancer cell fraction was estimated using PyClone^18^ (v 0.13.1). The variant allele frequencies, the closest integer copy number alterations, and the tumor purity of T1 and T2 from the same patients were used as input. If mutations were not presented or called in the sample, then the mutation allele frequencies were calculated as zero.

**REFERENCE**

1. Hou H, Yang X, Zhang J*, et al*. Discovery of targetable genetic alterations in advanced non-small cell lung cancer using a next-generation sequencing-based circulating tumor DNA assay. *Scientific Reports*. 2017;7(1): 14605.

2. Chen Y, Chen Y, Shi C*, et al*. SOAPnuke: a MapReduce acceleration-supported software for integrated quality control and preprocessing of high-throughput sequencing data. *GigaScience*. 2018;7(1): 1-6.

3. Li H & Durbin R. Fast and accurate short read alignment with Burrows-Wheeler transform. *Bioinformatics*. 2009;25(14): 1754-1760.

4. McKenna A, Hanna M, Banks E*, et al*. The Genome Analysis Toolkit: a MapReduce framework for analyzing next-generation DNA sequencing data. *Genome research*. 2010;20(9): 1297-1303.

5. Martínez-Jiménez F, Muiños F, Sentís I*, et al*. A compendium of mutational cancer driver genes. *Nature Reviews Cancer*. 2020;20(10): 555-572.

6. Jia W, Li H, Li S, Chen L & Li SC. Oviz-Bio: a web-based platform for interactive cancer genomics data visualization. *Nucleic Acids Res*. 2020;48(W1): W415-W426.

7. Liu M, Chen J, Wang X*, et al*. MesKit: a tool kit for dissecting cancer evolution of multi-region tumor biopsies through somatic alterations. *GigaScience*. 2021;10(5).

8. Alexandrov LB, Kim J, Haradhvala NJ*, et al*. The repertoire of mutational signatures in human cancer. *Nature*. 2020;578(7793): 94-101.

9. Rosenthal R, McGranahan N, Herrero J, Taylor BS & Swanton C. deconstructSigs: delineating mutational processes in single tumors distinguishes DNA repair deficiencies and patterns of carcinoma evolution. *Genome Biol*. 2016;17(1): 31.

10. Wala JA, Bandopadhayay P, Greenwald NF*, et al*. SvABA: genome-wide detection of structural variants and indels by local assembly. *Genome research*. 2018;28(4): 581-591.

11. Yin T, Cook D & Lawrence M. ggbio: an R package for extending the grammar of graphics for genomic data. *Genome Biol*. 2012;13(8): R77.

12. Mayrhofer M, DiLorenzo S & Isaksson A. Patchwork: allele-specific copy number analysis of whole-genome sequenced tumor tissue. *Genome Biol*. 2013;14(3): R24-R24.

13. Rajaby R, Zhou Y, Meng Y*, et al*. SurVirus: a repeat-aware virus integration caller. *Nucleic Acids Res*. 2021;49(6): e33-e33.

14. Li H. Minimap2: pairwise alignment for nucleotide sequences. *Bioinformatics*. 2018;34(18): 3094-3100.

15. Gouet P, Courcelle E, Stuart DI & Métoz F. ESPript: analysis of multiple sequence alignments in PostScript. *Bioinformatics*. 1999;15(4): 305-308.

16. Jia W, Xu C & Li SC. Resolving complex structures at oncovirus integration loci with conjugate graph. *Brief. Bioinform.* 2021;22(6): bbab359.

17. Carter SL, Cibulskis K, Helman E*, et al*. Absolute quantification of somatic DNA alterations in human cancer. *Nature biotechnology*. 2012;30(5): 413-421.

18. Roth A, Khattra J, Yap D*, et al*. PyClone: statistical inference of clonal population structure in cancer. *Nature methods*. 2014;11(4): 396-398.

**2 SUPPLEMENTAL FIGURES**


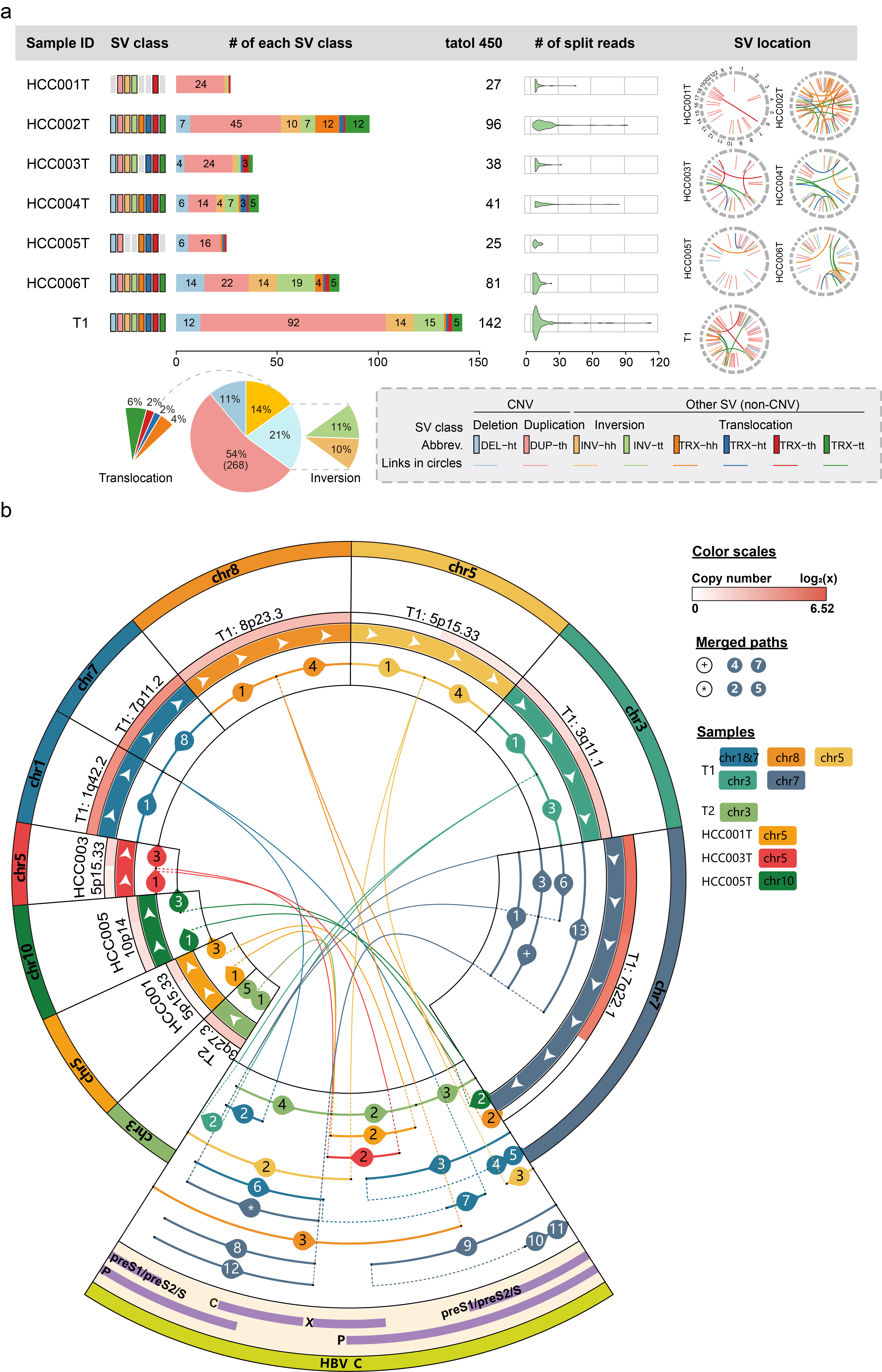


**Figure S1.** Characterisations of SVs and HBV integration of seven patients. (a) Somatic SVs of seven patients. Different colors represent different types of SVs. (b) Features of HBV integrated local haplotypes in five samples. Human genomic segments related to HBV integrated local haplotypes are shown as sectors with their relevant local haplotype path in sample-specific colors. Segments of local haplotypes are denoted by numbered concentric arcs. The numbers, starting from 1, indicate the order in which each arc is visited starting from the source segment of a local haplotype. Each special symbol shown in the "Merged paths" section in the figure legends denotes an arc that is visited more than once in the local haplotype, such as the '+' symbol for NO.4 and NO.7 arcs in the local haplotype of T1 sample. DNA copy number is displayed in gradient red color, with bilateral labels in relevant sample color. The HBV genome reference is AB014381.1 from the NCBI Nucleotide database. The detailed local haplotypes is shown in Figures 2(chr3 of T1 and T2), S2 (HCC001T, HCC003T, and HCC005T), S3 (chr1 and 7 of T1), S4 (chr5 and 8 of T1).


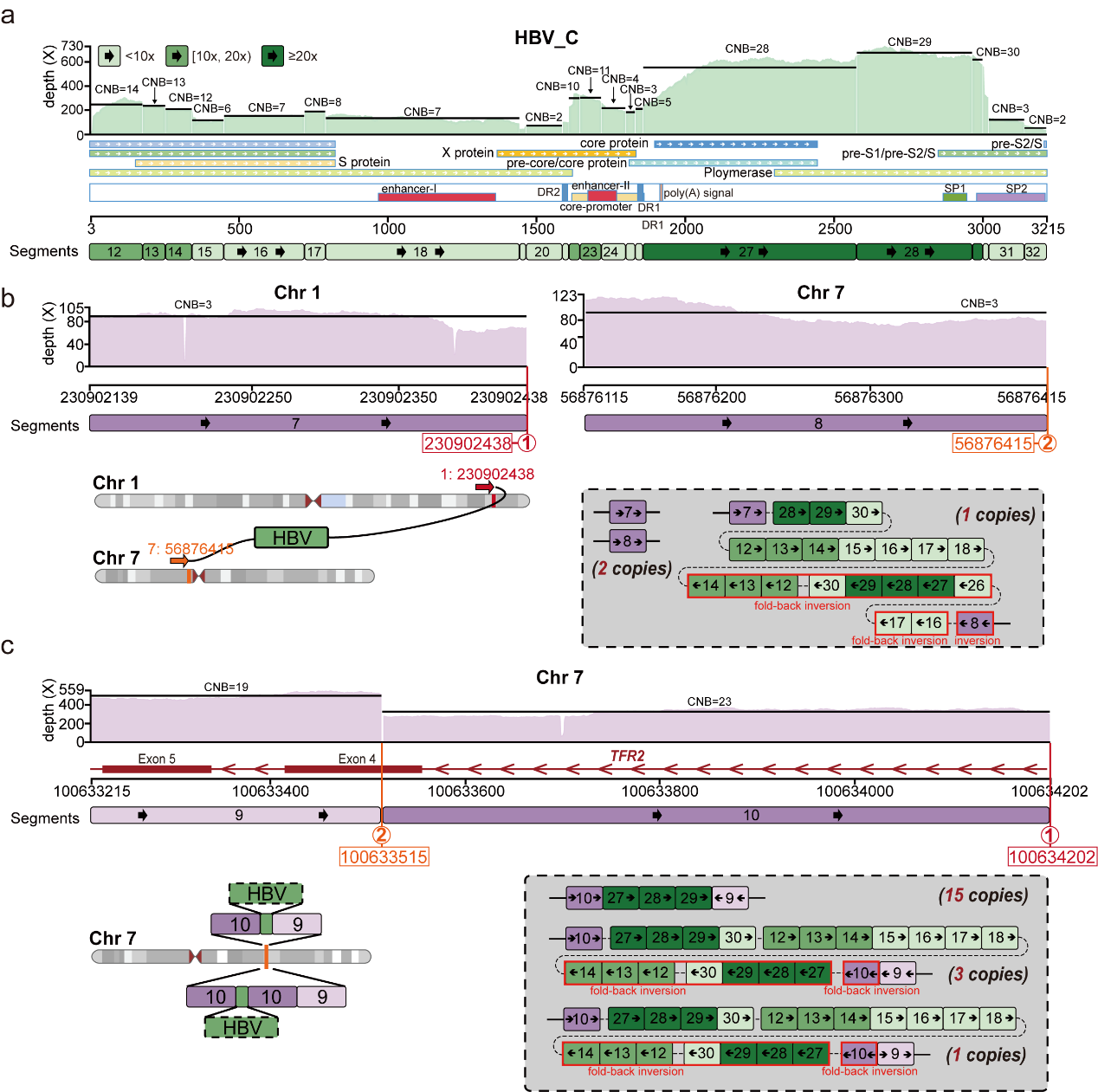


**Figure S2.** The local haplotype of HBV-integrated genomic region in chr1 and chr7 of T1. (a) Constructed HBV_C genome is segmented (12~32). The sequencing depth spectrum is displayed with balanced copy numbers of segments. Black lines denote the average depth of segments. (b) Human genomic regions of chr1 and chr7 flanking HBV integrations are divided into segments (7~8) by viral insertions. Breakpoints are noted by circled numbers. Top: Sequencing depth spectrum and balanced copy numbers of chr1 and chr7 segments. Bottom left: the fusion of chr1 and chr7 linked by an HBV bridge. The direction of the arrows represents the strand that the rearrangement segment was aligned to (right-facing is the forward strand). Bottom right: resolved alleles of local haplotype are indicated as colored segments connected string with copy times. The “fold-back inversion” means duplicated segments arranged head-to-head. (c) Local haplotype of HBV-integrated genomic region in chr7.


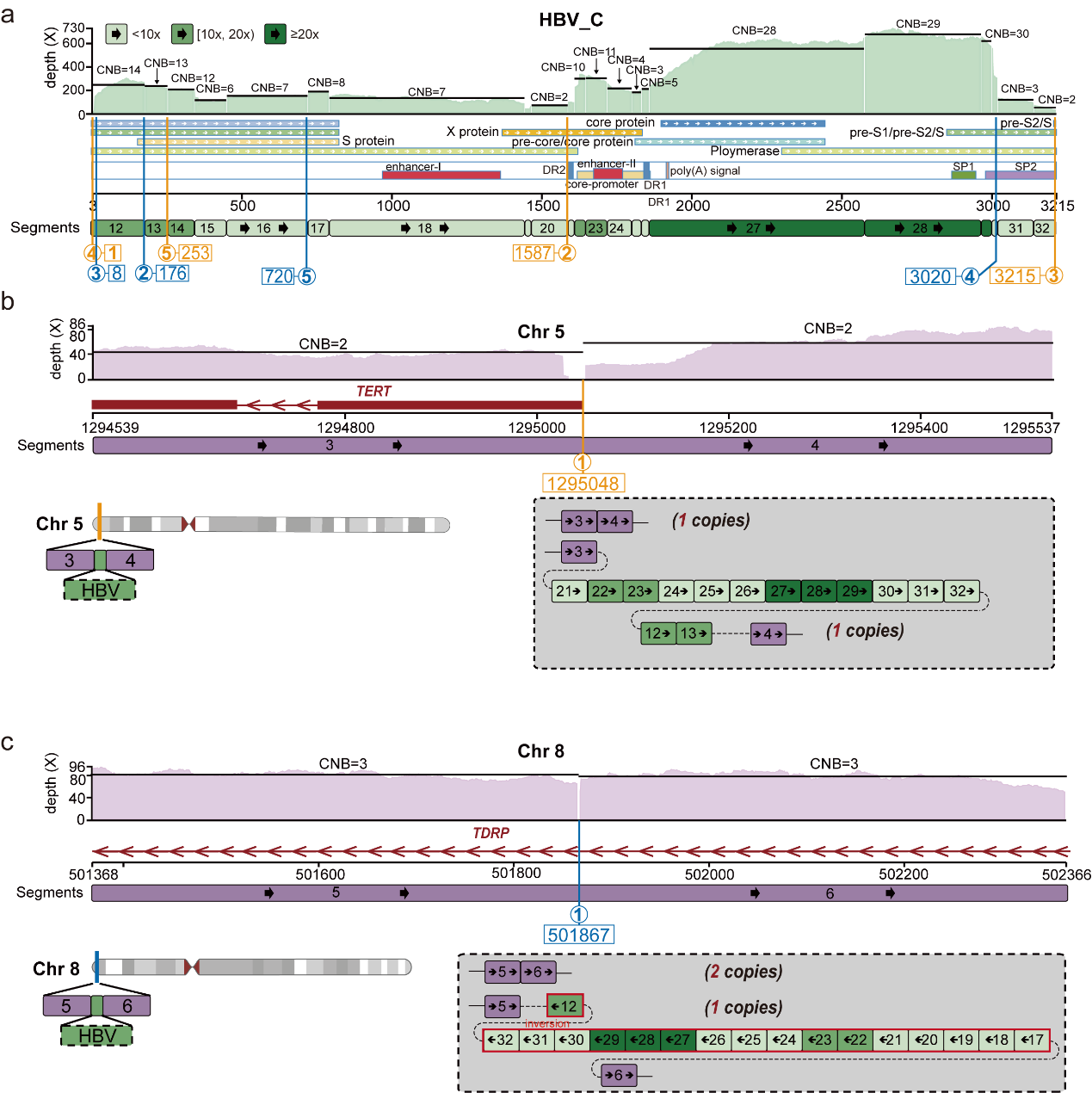


**Figure S3.** Presentative local haplotype at HBV integration loci on chr5 (*TERT*) and chr8 (*TDRP*) of the T1 sample.


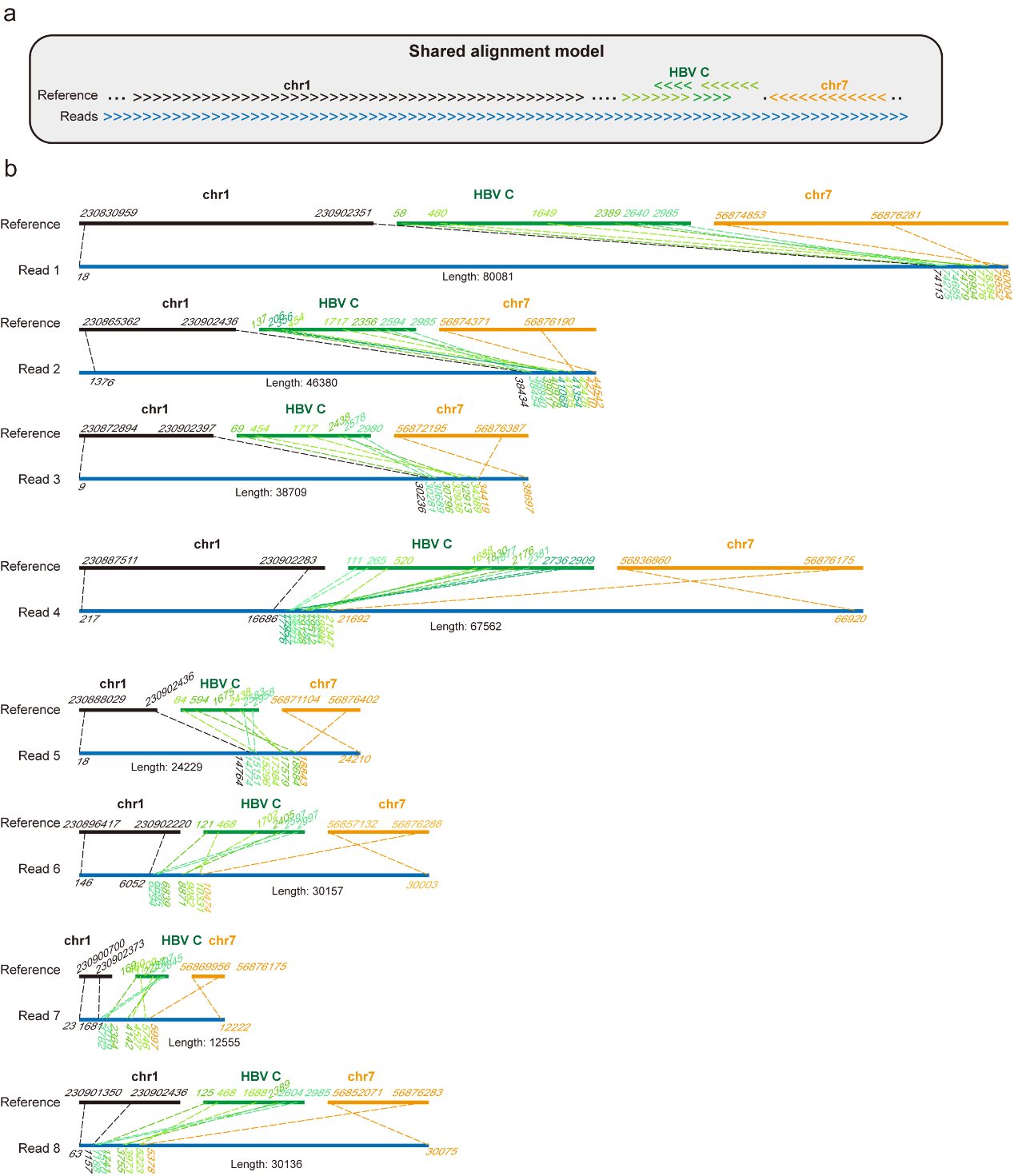


**Figure S4.** Eight long PacBio reads supported the fusion of chr1 and chr7 linked by an HBV bridge. (a) The shared alignment model of eight long PacBio reads to the reference. The same read was aligned to chr1, HBV C, and chr7 in turn (> is forward strand alignment; < is reverse strand alignment). (b) The alignments of eight reads to the reference. To improve readability, we unify the lengths of chr1, chr7, and HBV C reference. Their relative lengths can be observed from the aligned reads.


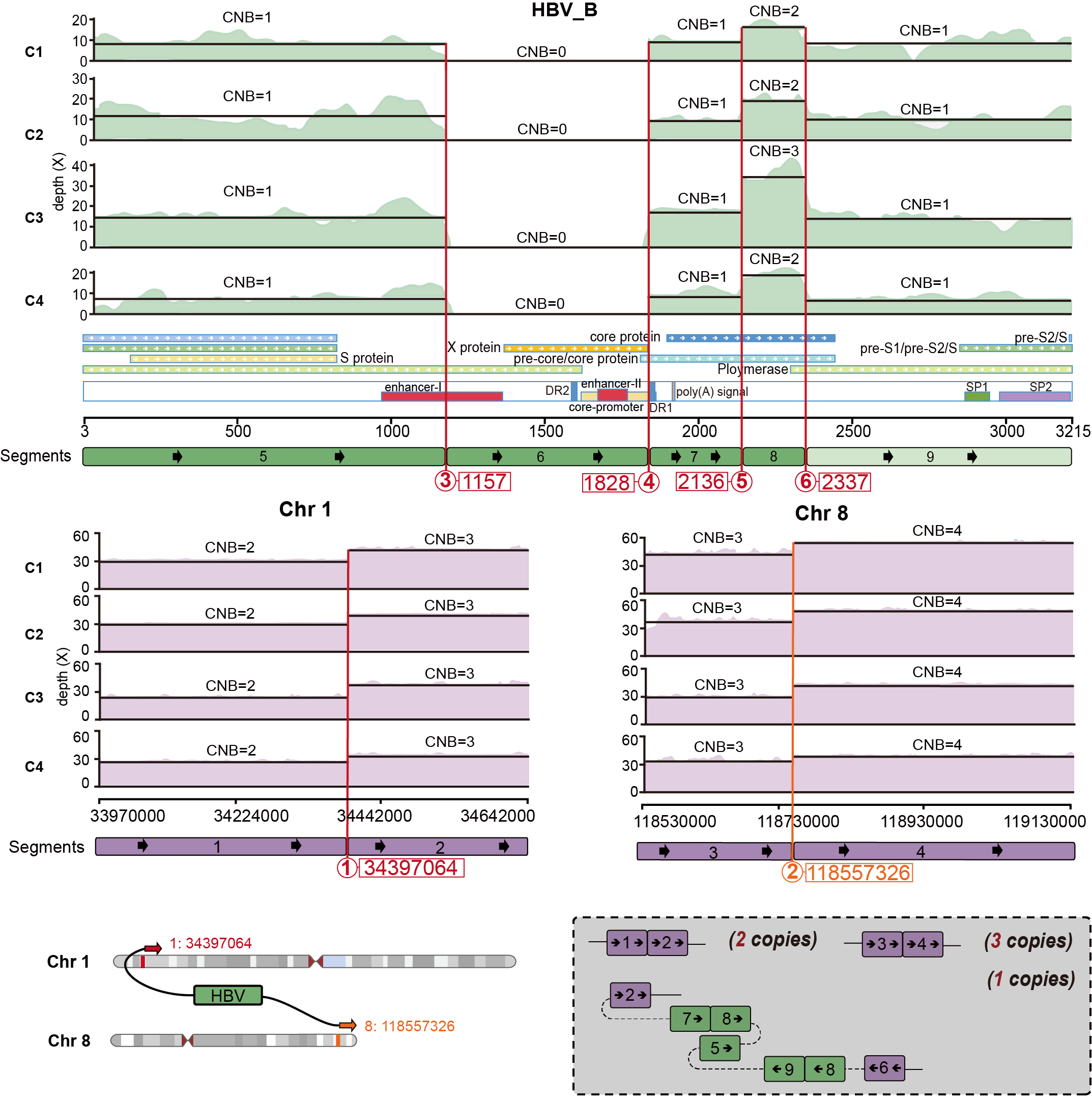


**Figure S5.** Presentative local haplotype at HBV integration loci on chr1 and chr7 of C1~4 samples. Constructed HBV_B genome is segmented (5~9). The sequencing depth spectrum is displayed with balanced copy numbers of segments. Black lines denote the average depth of segments. Human genomic regions of chr1 and chr8 flanking HBV integrations are divided into segments (1~4) by viral insertions. Breakpoints are noted by circled numbers.


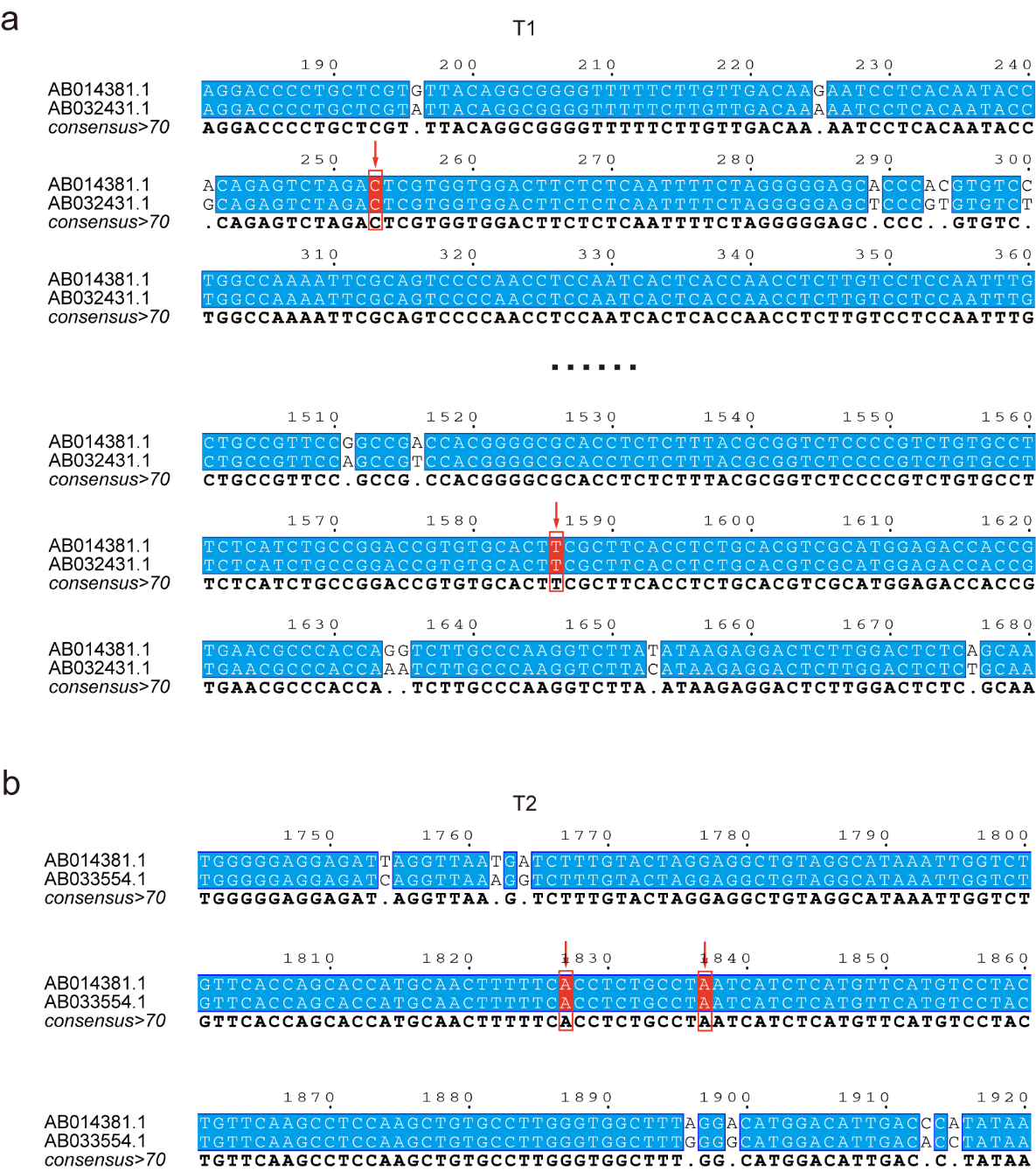


**Figure S6.** Sequence alignment between three HBV subtypes. (a) Sequence alignment between AB014381.1 (HBV C) and AB032431.1 (HBV E). (b) Sequence alignment between AB014381.1 (HBV C) and AB033554.1 (HBV B). HBV integration sites are denoted by red arrows.
